# Green Synthesis of Gold Nanoparticles Using Plant Extracts as Beneficial Prospect for Cancer Theranostics

**DOI:** 10.3390/molecules26216389

**Published:** 2021-10-22

**Authors:** Kaushik Kumar Bharadwaj, Bijuli Rabha, Siddhartha Pati, Tanmay Sarkar, Bhabesh Kumar Choudhury, Arpita Barman, Dorothy Bhattacharjya, Ankit Srivastava, Debabrat Baishya, Hisham Atan Edinur, Zulhisyam Abdul Kari, Noor Haslina Mohd Noor

**Affiliations:** 1 Department of Bioengineering & Technology, GUIST, Gauhati University, Guwahati 781014, Assam, India; kkbhrdwj01@gmail.com (K.K.B.); bijulipep@gmail.com (B.R.); arpitabarman532@gmail.com (A.B.); dorothybhatta@gmail.com (D.B.); 2 SIAN Institute, Association for Biodiversity Conservation and Research (ABC), Balasore 756001, Odisha, India; patisiddhartha@gmail.com; 3 Department of Biotechnology, Academy of Management and Information Technology, Khordha 752057, Odisha, India; 4 Malda Polytechnic, West Bengal State Council of Technical Education, Government of West Benga, Malda 732102, West Bengal, India; tanmays468@gmail.com; 5 Department of Food Technology and Biochemical Engineering, Jadavpur University, Kolkata 700038, West Bengal, India; 6 Department of Chemistry, Gauhati University, Guwahati 781014, Assam, India; bkcsat@gmail.com; 7 Department of Biotechnology, Motilal Nehru National Institute of Technology, Allahabad (Prayagraj) 211004, Uttar Pradesh, India; anku054@gmail.com; 8 School of Health Sciences, Health Campus, Universiti Sains Malaysia, Kubang Kerian 16150, Kelantan, Malaysia; edinur@usm.my; 9 Faculty of Agro Based Industry, Universiti Malaysia Kelantan, Jeli 17600, Kelantan, Malaysia; zulhisyam.a@umk.edu.my; 10 Haematology Department, School of Medical Sciences, Universiti Sains Malaysia Health Campus, Kubang Kerian 16150, Kelantan, Malaysia

**Keywords:** gold nanoparticles, AuNPs, green synthesis, anticancer, plants, therapy

## Abstract

Gold nanoparticles (AuNPs) have been widely explored and are well-known for their medical applications. Chemical and physical synthesis methods are a way to make AuNPs. In any case, the hunt for other more ecologically friendly and cost-effective large-scale technologies, such as environmentally friendly biological processes known as green synthesis, has been gaining interest by worldwide researchers. The international focus on green nanotechnology research has resulted in various nanomaterials being used in environmentally and physiologically acceptable applications. Several advantages over conventional physical and chemical synthesis (simple, one-step approach to synthesize, cost-effectiveness, energy efficiency, and biocompatibility) have drawn scientists’ attention to exploring the green synthesis of AuNPs by exploiting plants’ secondary metabolites. Biogenic approaches, mainly the plant-based synthesis of metal nanoparticles, have been chosen as the ideal strategy due to their environmental and in vivo safety, as well as their ease of synthesis. In this review, we reviewed the use of green synthesized AuNPs in the treatment of cancer by utilizing phytochemicals found in plant extracts. This article reviews plant-based methods for producing AuNPs, characterization methods of synthesized AuNPs, and discusses their physiochemical properties. This study also discusses recent breakthroughs and achievements in using green synthesized AuNPs in cancer treatment and different mechanisms of action, such as reactive oxygen species (ROS), mediated mitochondrial dysfunction and caspase activation, leading to apoptosis, etc., for their anticancer and cytotoxic effects. Understanding the mechanisms underlying AuNPs therapeutic efficacy will aid in developing personalized medicines and treatments for cancer as a potential cancer therapeutic strategy.

## 1. Introduction

The term “nanotechnology” refers to the design, development, and implementation of components that are 10–1000 nanometers in size. Nanotechnology is currently a multidisciplinary field that encompasses engineering, biology, chemistry, and physics [1,2]. The nanoparticle’s size, optoelectronic and chemical properties, along with their unique structural morphology, has attracted researchers toward various biomedical, and food-related implications [3,4,5,6]. Carbon nanotubes, polymeric nanoparticles, metallic nanoparticles, microbially synthesized nanoparticles, liposomes, and magnetic nanoparticles are among the most studied nanostructures for biomedical applications in cancer therapy and diagnosis [7,8,9,10,11,12,13,14,15,16]. Cancer, a disease under which the body’s healthy cells mutate from their normal state and divide uncontrollably, is recognized as a major medical challenge in the 21st century. One of the serious worries of scientists and physicians is the escalating incidence of cancer and the continuously growing mortality rate. Remarkably efficient cancer therapy and diagnosis are now regarded as one of the century’s toughest challenging obstacles in health and medicine. Despite significant advancements in drug development and delivery, their numerous side effects and poor specificity and sensitivity, which most often harm healthy tissues and organs, have been substantial impediments to their use [17]. On the other hand, the prevalence of tumour cells resistant to conventional cancer treatments such as chemotherapy, radiotherapy, and surgery, as well as causing numerous adverse responses and disappointing treatment outcomes, shows the critical need for the enhancement and development of antitumor therapeutic research that has the potential to target tumours and kill cancer cells effectively, having very few side effects [18]. Cancer nanomedicines, also known as magic bullets, have become widely utilized in cancer treatment because of their remarkable efficacy and safety. The use of nanoparticles in cancer treatment has substantially increased drug delivery to the target compared to conventional drug administration methods. It significantly enhances the safety and effectiveness of the most commonly utilised anticancer drugs. Effective targeting, delayed-release, prolonged half-life, and reduced toxicity are the significant benefits of nanomedicine and delivery systems [19]. Since the FDA approved the first nano formulated anticancer drug, Doxil Caelyx (Janssen) (Liposomal Doxorubicin (PEGylated)) in 1995, for the treatment of Ovarian and HIV-associated Kaposi’s sarcoma, the numbers of nanomedicines used to treat cancer has increased dramatically [20]. The US FDA and EMA currently approved various nanotechnology-based anticancer drugs, such as Myocet (Teva UK) (EMA 2000) (liposomal doxorubicin (non-PEGylated)) (treatment of metastatic breast cancer), DaunoXome (Galen) (FDA 1996, EMA 2018) (liposomal daunorubicin (non-PEGylated)) (HIV-associated Kaposi’s sarcoma), ONPATTRO Patisiran ALN-TTR02 (Alnylam Pharmaceuticals) (FDA 2018) (lipid nanoparticle RNAi) (Transthyretin (TTR)-mediated amyloidosis), and VYXEOS CPX-351 (Jazz Pharmaceuticals) (FDA 2017, EMA 2018) (liposomal formulation of cytarabine: daunorubicin) (acute myeloid leukaemia) [21]. Metallic nanoparticles are small metal particles that range in size from 1 to 100 nanometers (nm). Silver (Ag), aluminium (Al), iron (Fe), gold (Au), silica (Si), copper (Cu), zinc (Zn), manganese (Mn), cerium (Ce), titanium (Ti), platinum (Pt), or thallium (TI) have all been utilized in the synthesis of nanoparticles (NPs) [22]. Various metallic nanoparticles have been designed for the treatment and diagnosis of cancer. The most prominent anti-cancer nanoparticles are gold (Au), silver (Ag), zinc (Zn), and platinum (Pt) due to their size and shape-dependent tuneable optoelectronic properties [23]. Alloy NPs such as CoNi nanoparticles were successfully developed for cancer theranostics [24]. The unique size of the metal NPs impacts the toxicity in the human cancer cells and is size-dependent. The cytotoxicity of different-sized silver nanoparticles (AgNPs), when analysed, demonstrated that AgNPs with the size of 10 nm were more cytotoxic than those 20–80 nm size due to the high surface area, resulting in more efficient cell-particle interaction and increased intracellular silver bioavailability than larger NPs [25]. Tumour tissues are more permeable to NPs than normal tissues because of their leaky vasculature and inadequate lymphatic drainage. This facilitated the synthesized NPs being more permeable to tumour tissues, enabling NPs to easily penetrate cancerous cells and kill them. Furthermore, the presence of hydrophilic molecules on the surface of metal nanoparticles increases their solubility and half-life by protecting them from macrophage-mediated absorption, allowing them to stay in the systemic circulation longer [26,27]. Gold nanoparticles (AuNPs) are becoming promising cancer therapeutic and diagnostic metal NPs that attract researchers due to their unique physiochemical properties such as stability, biocompatibility, high thermal activity, optical, electrical, high surface area to volume ratio surface chemistry, and multifunctionalization, etc. By fine tuning the components and concentrations, AuNPs can be easily manufactured into various forms and sizes [28,29,30]. AuNPs have also shown significant advancement in treating inflammatory diseases and bacterial infections [31,32].

**Figure 1:**
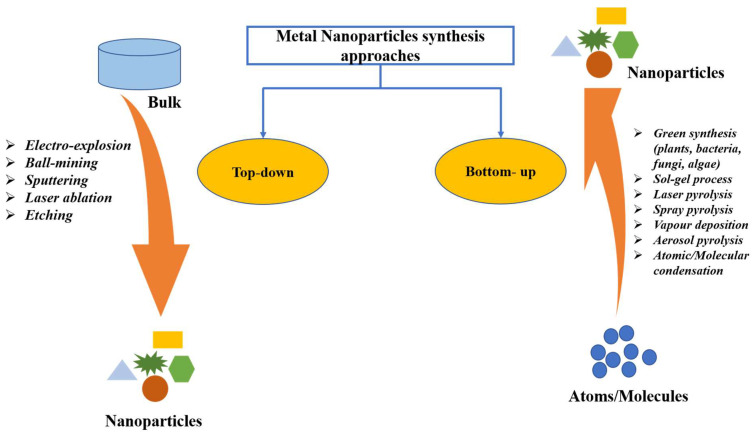
Different approaches of metal nanoparticles synthesis.

Green synthesis is regarded as a promising method for NPs synthesis because of the utilization of cost-effective and non-hazardous raw materials [33]. This method of NPs synthesis is sustainable with great therapeutic efficacy, reduced toxicity, targeted binding, and site-specific delivery. Additionally, this does not affect human health and the environment [34,35]. As shown in Figure 1, NPs are typically synthesized using two approaches: top-down and bottom-up. The most means of NPs synthesis are found in the bottom-up approach, in which NPs are made from simpler molecules [36]. Due to economic cost, the plant-based green synthesis methodology is one of the best choices for the large-scale generation of AuNPs with well-defined size and morphology [37]. Plants are preferred candidates for NPs synthesis over microorganisms, such as fungi and bacteria, since plants do not require the complex process of maintaining microbial cultures [19].

This article aims to provide an overview of green synthesized AuNPs, including their characterization methods and applications in cancer therapy and diagnosis. Anticancer properties of AuNPs are detailed, along with their putative mechanisms of action on various cell lines, based on the literature. Important therapeutic and future challenges of AuNPs in terms of anticancer were reviewed before the article was concluded.

## 2. Properties of Gold Nanoparticles

To synthesize stable nanoparticles (NPs), gold (Au) is regarded as suitable metal. Most of the physical properties of inorganic nanoparticles are found to be dependent on the size and shape of the NPs. The gold nanoparticles (AuNPs) have wide applications in different fields due to their specific optical and physical properties. AuNPs possess significant properties, such as (a) small size (1–100 nm), (b) physical and chemical properties based on size, shape, and composition, (c) excellent robustness, (d) qualitative and quantitative target binding ability, etc. [38].

### 2.1. Shape and Size

There are various methods for the synthesis of AuNPs having different shapes and sizes. The size and shape of AuNPs are the two primary parameters that control the chemical, physical and electrocatalytic properties of gold nanoparticles. Metal nanoparticles in the size range of 1–10 nm have size-dependent properties compared to bulk materials [39]. There are very few methods for the production of AuNPs with uniform sizes. Michael Faraday introduced the two-phase system to prepare AuNPs by reducing gold salt by phosphorous in carbon disulphide for the first time. Researchers have developed a popular method for synthesising smaller AuNPs. They reduced gold salt by sodium borohydride in the presence of the capping agent dodecanethiol [40]. They confirmed the size of the NPs in the range 1–3 nm using HRTEM (High-Resolution Transmission Electron Microscopy). It is possible to control the size of NPs by varying the concentration of thiols. G. Frens agreed with Turkevich’s data that reduction of gold chloride salt using sodium citrate solution is an efficient method to prepare monodispersed gold nanoparticles of different diameters. By changing the ratio of reactants, independent nucleation and growth of metal nanoparticles with different diameters can be achieved [41,42]. The size of AuNPs are tuneable through the alteration of gold precursor and reducing the power of reducing agents. It is observed that strong reducing agents, such as NaBH_4_ offer the synthesis of small size AuNPs and weaker reducing agents, such as citrate, resulted in comparatively larger NPs. Researchers have investigated the effect of chloride ion concentration on the size of AuNPs via citrate reduction. They find that size of gold NPs are caused by the aggregation of gold NPs induced by chloride ions [43]. In a typical synthesis, chloroauric acid (HAuCl_4_) is reduced by 5% sodium citrate solution at room temperature. The chloride ion concentration is varied using different concentrations of NaCl solutions. They performed the same experiment using NaBH_4_ as a reductant, and the interesting results are placed in Table 1. The table includes the data for the effect of different concentrations of NaCl on the size of AuNPs in the presence of different reactants such as sodium citrate and sodium borohydride, respectively. With an increase in NaCl concentration from 1 to 20 mM, the UV-visible absorption maxima (max) are also shifted towards higher wavelengths that indicate an increased size of AuNPs.

**Figure 2:**
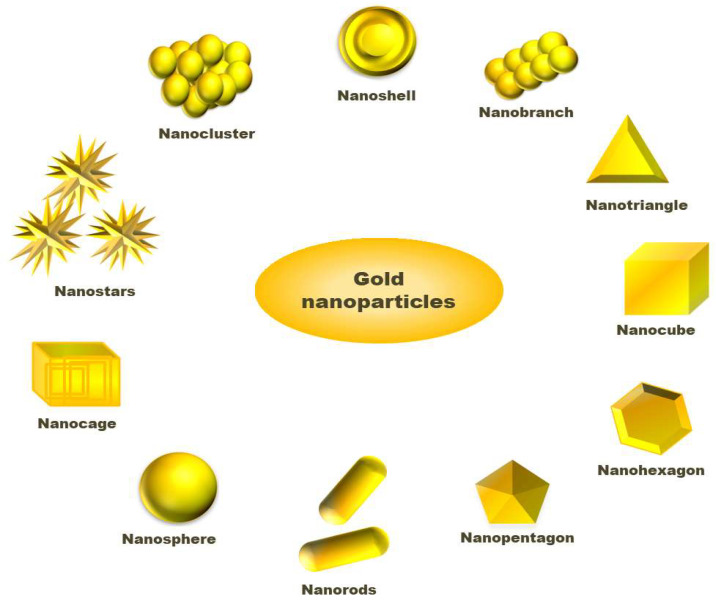
Different shapes available for gold nanoparticles.

Solvents used in the synthesis of NPs play an important role as either interaction between nanoparticle surface and solvent molecule or solvent and ligand molecules direct its final size and morphology [44,45]. Scholars have challenged the two-phase synthesis of NPs by Brust and co-workers and developed a single-phase synthesis of monodispersed gold nanoparticles using borane complexes as a reductant in organic solvents. In a typical process, AuPPh_3_Cl (0.25 mmol) and dodecanethiol (0.125 mmol) is mixed with benzene, and 2.5 mmol solution of the tert-butylamine-borane complex is added as reductant that forms 6.2 nm size gold nanoparticles [46]. In a modified Stucky’s method, Song and coworkers have prepared mono dispersed AuNPs from the reaction of AuPPh_3_Cl with an amine borane complex tert-butylamine borane (TBAB) having thiol ligand. In the absence of thiol, the AuNPs are found to have polydispersity, while in the presence of thiol, the synthesized NPs are monodispersed, having a size of 5.0 nm ± 0.4 nm [47]. To synthesize large size AuNPs, the seeded growth method is preferred. In this method, small size gold nanoparticles have been synthesized that act initially as seeds for the growth of larger gold nanoparticles. Then, separation of nucleation and growth of the NPs, we may increase the size of AuNPs up to 300 nm [39,48]. Stanglmair and co-workers have reported synthesizing monodispersed AuNPs with 20 nm average diameter in size via the seeded growth method. They synthesized gold nanoparticles of 9 nm size, in toluene as solvent and oleylamine as reductant cum stabilizing agent, were further used as a seed to produce AuNPs of 20 nm average size. Researchers have reported the synthesis of spherical shape AuNPs from H[AuCl_4_]·3H_2_O as a precursor, ascorbic acid as reductant and sodium citrate as a stabilizer. In the first step, they were able to synthesize NPs with 30 nm size, which then acts as a seed for the production of AuNPs, having 69 nm and 118 nm sizes and even further growth [49]. The report shows that AuNPs with different sizes and shapes, such as long nanorods, short nanorods, cubes and spheres can be prepared via reversible flocculate formation surfactant micelle-induced depletion interaction. To obtain different shapes of NPs, the tuning of surfactant concentration and extraction of flocculates from the sediment are important steps [50]. Jianhui Zhang, with his co-workers, has investigated the shape-selective synthesis of AuNPs with controlled size and different shapes, such as hexagons, belts, rods, triangles, octahedrons and dumbbells. In the process, water molecules are attached with poly(vinylpyrrolidone) (PVP) and *n*-pentanol to form a two-phase system of water/PVP/n-pentanol (WPN). PVP can act as a reductant and stabilizer where the presence of water can modify the reducing ability of PVP. However, they utilized PVP as a capping agent rather than a reductant. They have observed the region-selective distribution of water and PVP in the WPN system, which offers kinetically controlled growth of novel AuNPs nanostructures [51]. Researchers have studied the effect of temperature on the size of AuNPs by synthesizing gold nanoparticles varying temperature using tetraoctylammonium bromide (TOAB) as a stabilizer. The room temperature synthesis confirms AuNPs with an average size of 5.2 nm having a spherical shape. When the annealing of the AuNPs synthesis was performed at 100 °C for 30 min, a drastic change in the shapes of the gold nanoparticles was observed. The sizes of nanoparticles change from 5.2 nm to 6 nm, bearing shapes such as hexagons, pentagons, and squares under HRTEM observation, whose corresponding three-dimensional shapes are cuboctahedron, icosahedron, and a cube, respectively. On annealing, at 200 °C, the morphology, as well as size of AuNPs, changed drastically. The HRTEM shows the average size of the NPs to be 15 nm with different shapes, such as hexagon, triangle and pentagons. Similarly, nanoparticles and nanocubes are obtained when annealing is performed at 300 °C [52]. An interesting process, called the dewetting process, has attracted the researcher in the synthesis of nanoporous AuNPs. In this method, Au/Ag bilayer alloy film is initially produced, where AuNPs are much smaller in comparison to silver nanoparticles (AgNPs). Then, AgNPs are removed by treating the Au/Ag bilayer in 65 wt% HNO_3_ solution at 21 °C, called dealloying. After dealloying, Au (5 nm)/Ag (20 nm) bilayer AuNPs with 274 nm are found, while Au (10 nm)/Ag (20 nm) bilayer-formed AuNPs have a diameter of 307 nm [53]. Researchers have reported the synthesis of cap-shaped AuNPs with 110 nm size by evaporating gold adsorbed on polystyrene [54]. The atom-transfer radical polymerisation (ATRP) is a technique used by scholars to prepare monodispersed nanoparticles that might be useful for synthesizing NPs of other precursors [55]. Researchers have prepared gold nanoclusters in the size range 7–20 nm having positive and negative charges in presence of polyamidoamine dendrimers (PAMAM) or sodium citrate [56]. Luca and co-workers have synthesized gold nanostar (AuNS) from HAuCl_4_ as a precursor using hydroxylamine as a reductant above pH 11 maintained by HaOH solution. AuNS are formed in the pH range 12–12.5 where below pH 11, no reduction occurs to Au (III) species. Thus, pH plays an important role in the size and morphology determination of AuNPs [57]. The graphical abstract presented in Figure 2 tries to include varieties of available shapes for AuNPs.

### 2.2. Optical Properties

Nanoparticles possess excellent optical properties that are different from individual molecules and bulk metals. The optical properties of AuNPs related to surface plasmon resonance (SPR) are one of the reasons behind the vast success of AuNPs in nanoscience and technology [58]. As NPs are exposed to light, the oscillating electromagnetic field of light automatically induces collective coherent oscillation in the free electrons present in the conduction band of NPs. This eventually results in the charge separation that forms a dipole oscillation in the electric field of light. The amplitude of this oscillation reaches the zenith of maximum at a particular frequency known as surface plasmon resonance (SPR). The extent of SPR can be measured using a UV-visible spectrophotometer as the SPR absorbance for nanomaterials is much stronger than other metals. As per Mie theory, the SPR band intensity and wavelength depend upon factors, such as metal type, size, shape and structure of NPs, composition, and dielectric constant of the medium [59]. Researchers have used the photoacoustic technique (PA) to study the optical absorption properties of AuNPs with different shapes and sizes synthesized by them. They synthesized gold nanorods and nanospheres via seed-mediated growth techniques. For gold nanorods, the PA spectra are found to split into two modes, namely transverse and longitudinal. It is interesting to note that gold Nanospheres having size ~20 nm in diameter have a characteristic strong PA absorption band centred at ~522 nm while for nanorods, there was observed a two-band centred at ~522 nm and ~698 nm for transverse and longitudinal SPR. The gold nanoparticles of size < 2 nm in diameter do not show such absorption [60]. Researchers have reported a green synthetic method for AuNPs using starch as a reductant as well as capping agent. The report revealed second-order nonlinear optical (NLO) properties studied using continuous wave (CW) He-Ne laser beam technique at wavelength 632.8 nm. The non-linear refractive indices of AuNPs are obtained from a z-scan in the order of 10^−7^ cm^2^/W [61]. Researchers have reviewed various aspects of size-dependent SPR of AuNPs. The investigation shows that branched nanoparticles, also known by different other names, such as multi-pod, lumpy, star-shaped, sea urchin-like, etc. are not highly monodispersed as compared to other shapes. The longitudinal SPR (LSPR) of such branched nanostructure is well understood with the help of the plasmon hybridisation model (PH). This method calculates the LPSR of complex structures, assuming it to be the result of LPSR of simpler structures [62]. Optical transmission spectroscopy can be employed to study surface plasmon excitation for two identical interacting spherical AuNPs. Researchers have studied SPR for three pairs of AuNPs with sizes 450 nm, 300 nm and 150 nm in interaction. It is found that, with a decrease in the inter-particle distance, red shifts in SPR are observed, while blue shift is found for orthogonal polarisation [63]. AuNPs can enhance the Raman signal from 10^6^ to 10^15^ when exposed p monochromatic light. This phenomenon is called the surface-enhanced Raman scattering (SERS) technique that can be applied to distinguish tumour cells, mark tumour cells, or monitor tumour metabolism. AuNPs contain radioactive atoms that help in achieving desired radioactivity for treatment. Due to the high molar coefficient of AuNPs, they can be employed in the nanomolar level colourimetric analysis, as there is the advantage of the colour change of the AuNP-induced plasmon from red to blue, purple or grey after aggregation. The LPSR of AuNPs can act as a fluorescence quencher and fluorescence enhancer based on the distance between the fluorescence probe and AuNPs. The fluorescence resonance energy transfer (FRET) is considered a primary reason for fluorescence quenching by AuNPs. Aside from this, AuNPs can absorb photons to convert the light energy of a photon to kinetic energy. When light falls on AuNPs, the moving electrons are scattered by photons. A part of kinetic energy transformed to vibrational energy that was eventually expressed in the form of heat in the lattice. This is known as a photothermal effect. On photoexcitation, AuNPs can absorb energy and go to an excited state and then transfer energy to neighbouring molecules to act as photosensitizers similar to other organic photosensitizers or molecular oxygen [64].

### 2.3. Electrical Properties

The semiconductor industries believe that complementary metal-oxide semiconductors (CMOS) will reach their functional limits within 10–15 years. Then, nanomaterials or molecular assemblies on the nanometer level will occupy the space. Promising concepts developed in recent years include single-electron devices that retain their scalability up to molecular level. Individual charge carriers can be handled by exploiting Coulombic effects in metallic single-electron devices with tunnel junctions with micrometer size. Such a field is termed single electronics (SE). The AuNPs have attracted the research’s attention in the approach to bridge the gap between CMOS and true atomic scale in the future [65]. In the case of nanoelectronics, monodispersed nanoparticles have a potential lot. Metal nanoparticles having a diameter < 2 nm are required for such devices to achieve the Coulomb blockade effect at room temperature [66]. Researchers have synthesized AuNPs through a green synthetic procedure using *Solanum nigrum, Ricinus communis* and *Morus nigra*, etc., extract as reducing agents. They performed experiments to evaluate the effect of adding AuNPs in the DC electrical conductivity and found that, with the increased addition of AuNPs, the DC electrical gradually increases [67]. Researchers have studied the size dependency of electronic properties of AuNPs nanoclusters up to 14 atoms through density functional theory and agree that the energetic and electronic properties of AuNPs nanoclusters depend on the size structures NPs [68]. Scholars have investigated the solvent-switchable electronic properties of gold nanoparticle/hydrogel composite. They have prepared a crosslinked polyacrylamide gels on Au-wire electrodes through the electro-polymerisation of acrylamide along with ZnCl_2_ and *N*,*N*′-methylenebisacryamide. The “breathing” mechanism is adopted to introduce AuNPs into the polymer. It is found that the polymer used to get swollen when placed in water and get shrunken in acetone. The X-ray photoelectron spectroscopy (XPS), atomic absorption spectroscopy (AAS), quartz crystal microgravimetry (QCM), and Faradic impedance spectroscopy are used to know the inclusion of AuNPs in the polymer that shows a decrease in the swelling of the polymer with an increase in the AuNPs load. As the Au-polymer swells, it exhibits a resistance of 40 KΩ; while shrunken in acetone, it bears a resistance of 0.4 KΩ. Thus, it shows solvent switchable electrical properties [69]. Structural dielectric capacitors (SDCs) on doping with AuNPs shows enhanced electrical conductivity. They can improve both electrical as well as mechanical properties of carbon fibre reinforced polymer (CFRP)-based electrodes used in SDCs through AuNPs doping via epoxy matrix phase. AuNPs in the size range ~2–5 nm in diameter are doped in different weight percentages in the range 0.025–1.0%. In this experiment, graphene oxide (GO) film is a dielectric layer that separates two CFRP electrodes in SDCs. It is found that the electrical conductivity of AuNPs doped CFRP electrodes is 15–250% higher than bare CFRP electrodes based on AuNPs percentage [70]. Similarly, scholars have reported the enhancement of electrical conductivity of carbon nanotube networks used as conductive fillers in a nanocomposite through AuNPs doping [71]. Silicon has semiconductor properties, and availability is considered a feasible option in the devices where biological responses are recorded as electrical signals. However, processing silicon materials as porous ones reduces the level of conductivity due to oxidation. This problem is resolved by embedding AuNPs into silicon-based materials through sputtering techniques. The gold NPs doped-silicon fibre composite shows more excellent conductivities at higher AuNPs concentrations with silicon nanofibers of smaller sizes [72].

## 3. Green Synthesis of Gold Nanoparticles (AuNPs) from Plants

**Figure 3:**
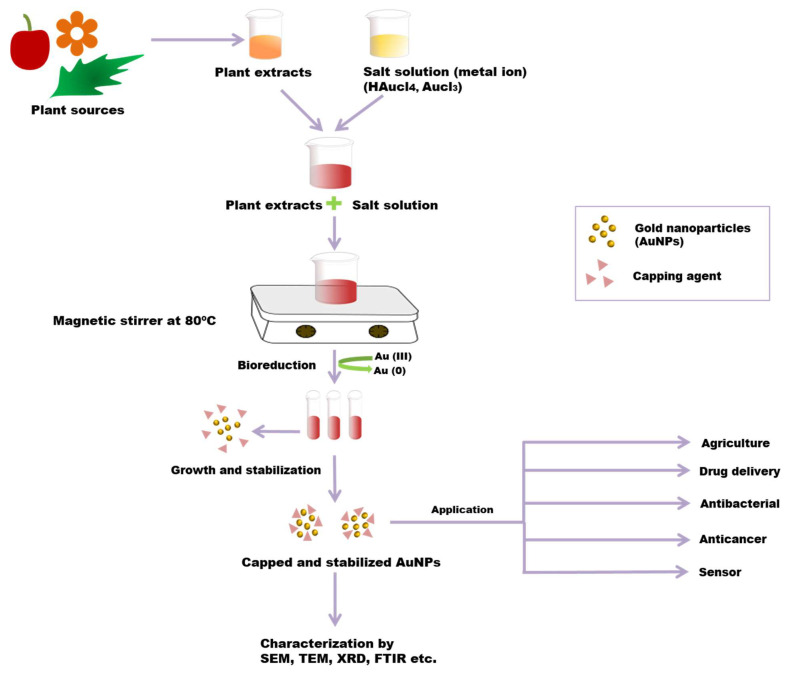
Green synthesis of AuNPs from a plant. Plant extract and metal salt solution HAuCl_4_ were mixed. After that, the resultant solution is centrifuged, which results in the bio reduction of metallic ions to AuNPs. Phytochemicals act as reducing, as well as stabilizing/capping, agents in this process. The resultant AuNPs are characterized by using SEM, TEM, FTIR, XRD, etc.

Different physical and chemical synthesis protocols have been well known for the biosynthesis of AuNPs. However, most of those protocols were not well accepted due to toxic chemicals and elevated temperature in the synthesis process. They may be harmful to human beings and the environment [73,74]. The most common biosynthetic method is the extracellular nanoparticle production method [28]. The green synthesis of gold nanoparticles has been reported using plant tissues, bacteria, fungi, actinomycetes, etc. (Figure 3) [75]. However, the green synthesis of AuNPs from the plant is an eco-friendly approach. In the biosynthesis of AuNPs from the plant, different plant parts (leaf, bark, stem, root, etc.) are used as sources chopped into small pieces and boiled in distilled water to obtain the extract. By filtration and centrifugation, the extract can be purified. For metal salt solution HAuCl_4_, AgNO_3_ generally is mixed with plant extract at room temperature [33,75]. Plant extracts contain various metabolites or organic compounds (alkaloids, flavonoids, proteins, polysaccharides, cellulose, and phenolic compounds) and secondary metabolites, which are utilized for nanoparticle synthesis [76]. These can involve the bio reduction of metallic ions to NPs and act as stabilizing agents [77]. Plant extracts contain proteins that have functionalized amino groups (–NH_2_) that can actively participate in the reduction reaction of AuNPs [68]. The functional groups (such as –C–O–C–, –C–O–, –C=C–, and –C=O–) present in phytochemicals, such as flavones, alkaloids, phenols, and anthracenes involve the generation of AuNPs. In this phenomenon, no external stabilising/capping agents are used because different phytochemicals act as reducing and stabilising/capping agents for the extracellular biosynthesis of AuNP, replacing the toxicity of chemicals such as sodium borohydride (NaBH_4_) [78]. The bio reduction mechanism involves reducing metal ions from their mono or divalent oxidation state to a zero-valent state. After that, the nucleation of the reduced metal atoms takes place [79]. Ultimately, the metallic salt solution containing extract is reduced into Au^3+^ to Au^0^, and the synthesis of AuNP proceeds within minutes to hours using a one-pot, single-step and eco-friendly method [80]. Due to the presence of a variety of phytochemicals in plant extract, no particular mechanism for this synthesis process is reported. The variation in composition and concentration of reducing agents in plant extracts is responsible for different sizes, shapes, and morphological nanoparticle synthesis [81]. Researchers have reported that the size and morphology of nanoparticles can be expected to be different by changing the synthesis parameters, including pH, metal salt, pH, temperature and reaction time [82].

Synthesized AuNPs were initially identified in the change in reaction colour (formation of red colour) through UV-vis spectrophotometer analysis. DLS, XRD and SAED confirmed the crystalline structure of gold nanoparticles, and the size, shape and distribution of nanoparticles were visualized by TEM image. Based on FTIR analysis, it can be confirmed that functional groups such as –C–O–C–, –C–O–, –C=C–, and –C=O are the capping ligands of the nanoparticles [83]. Different plant parts are used as a source for AuNP biosynthesis. Some green synthesized AuNPs from various plant parts are listed in Table 2.

### 3.1. Advantages and Limitations of the Synthesis Methods

Chemical methods for the synthesis of AuNPs have many limitations, which include environmental and biocompatibility concerns. Some of the chemicals used in the synthesis of gold nanoparticles during chemical synthesis can affect our environment and are the cause of risks for administering them into living organisms, thus limiting the biological applications of such AuNPs [121]. Therefore, various biological methods have been devised for the synthesis of AuNPs to limit these concerns. The green synthesis of AuNPs is a simple, safe, dynamic and facile process as its protocol follows a moderate environment without extreme temperatures or pressures. It is a cost-effective, rapid, environmentally benign, and biocompatible process, thus safe for clinical research. AuNPs are being synthesized through different physicochemical methods [122]. However, biogenic reduction of the gold salt to synthesize AuNPs is an inexpensive, eco-friendly and safe process. Neither toxic chemicals, such as sodium borohydride NaBH_4_, are used, nor are any contaminants or harmful/dangerous by-products produced in this process. Moreover, a considerable number of AuNPs of controlled size and morphology can be easily synthesized. Their stability and reduction potential are attributed to bioactive molecules present in these biological resources. Green synthesized AuNPs application improves the diagnosis and treatment of many human diseases [78]. Out of many biological resources, plant extracts are reported to be a more beneficial resource. Various plant metabolites, such as alkaloids, polyphenols (catechin, flavones, taxifolin, catechin and epicatechin, and phenolic acids), alcoholic compounds, glutathiones, polysaccharides, antioxidants, organic acids (ascorbic, oxalic, malic, tartaric, and protocatechuic acid), quinones, proteins, and amino acids are involved in the formation of NPs by the reduction of metal ions. FT-IR and HPLC tests were used to indicate the presence of these capping agents in the synthesized NPs [76]. Therefore, in this prospect, using plant sources for Au NPs synthesis can open new horizons in future. The primary goal of green nanotechnology is to curtail forthcoming environmental and human health risks associated with the use of nanotechnology products and inspire the substitution of existing products with a more environmentally friendly nano-product. AuNPs synthesis through this green method can contribute to other fields such as green photocatalyst, drug delivery, anti-microorganism, adsorbent, detector, and green separation science and technology [77]. The green synthesis of AuNPs from bacteria is a slow process, so the synthesis process can take a long time, comprising hours and even days. Green synthesis from fungi is better than the previous one, as fungi produce a large number of proteins and reactive compounds. As a result, the reaction process can be scaled up using fungi as a source [116,123]. Although green synthesis of AuNPs from the plant has many advantages, the limitation of using a plant as a source for the synthesis of AuNPs is that the identification of reactive components is difficult as plant biomass comprises a large number of organic components [124,125]. Biomolecules in the plant source contain various functional groups, which can play an essential role in synthesizing AuNPs, but different biomaterials show different reducing abilities. So, it is crucial to first determine their reducing ability before using them in the synthesis reaction [126,127].

### 3.2. Plant-Based Synthesized Gold Nanoparticles as Anticancer Agents

Increasing nanotechnology applications have gained broad attention in various sectors in recent years, but not restricted to medical, cosmetics, medical devices, electrical and electronic, drugs, food and packaging [128]. The most promising approach in nanotechnology is to develop nanomaterials for use in healthcare. In recent years, it has been observed that nanomaterials, such as gold nanoparticles (AuNPs), are of great interest to humans due to their wide range of uses in agriculture, remediation, medicine, health aspects, industry, pharmaceuticals, etc. [129]. Preliminary studies have shown that green synthesized AuNPs have various biological functions, such as antimicrobial, antiviral, anti-inflammatory, antioxidant and anticancer activity. In recent years, the use of plant-derived AuNPs has brought significant advances in cancer diagnosis and treatment, although some work in this area began mainly a few decades ago [128]. Notably, studies have demonstrated the usefulness of AuNPs as anticancer agents, in addition to photothermal agents, contrast agents and drug carriers. However, there are no previous literature reports on the molecular mechanism of tumour inhibition mediated by plant AuNPs. A recent resurgence of the anticancer effects of AuNPs from plant extracts has taken great strides so far. Despite these encouraging advances, more research is needed to understand the molecular consequences in cancer therapy, such as cellular toxicity, mitochondrial toxicity, apoptosis, necrosis and the production of reactive oxygen species (ROS). Several studies and reviews have been undertaken to investigate the anti-cancer potential of green synthesized AuNPs from different plant species. Scholars have reported on the green synthesis of AuNPs from several important plants and their applicability in various biomedical applications [130]; in this context, other authors have also proposed the implication of biosynthesized AuNPs in various applications. Researchers have reported on the aqueous and ethanolic extract of *Taxus baccata* synthesized nanostructure AuNPs. They were characterized by different techniques, such as UV–Vis spectroscopy, TEM, SEM and FT-IR. The MTT assay was performed to examine the anticancer activity of colloidal AuNPs on cell lines, such as Caov-4, MCF-7 and HeLa. In addition, an in vitro experiment on cell exposure to *T. baccata*-mediated AuNPs confirms the caspase-independent death program as an anti-cancer mechanism with increased efficacy for cancer therapy. This issue has been explored using flow-cytometry and real-time PCR [37]. Many plants (*Camellia sinensis*, *Coriandrum sativum*, *Mentha arvensis*, *Phyl-lanthus amarus*, *Artabotrys hexapetalus*, *Mimusops elengi*, *Syzygium aromaticum*) were described by Priya and Iyer, which showed that the green synthesized AuNPs have anticancer activity against the human breast cancer cell line, i.e., MCF7 and found that AuNPs at a minimum concentration of 2 μg/mL for cancer therapy are as effective as standard drugs. Moreover, the increase in the nanoparticle concentration is directly proportional to the effectiveness against cancer [131]. The increasing demand for biosynthesized gold nanoparticles has been greatly facilitated in medical applications, particularly in targeted drug delivery, one of the most recent advances in nanotechnology. Further studies have shown that the use of the *Dysosma pleiantha* rhizome can improve cancer therapy, which has been proven experimentally by tracking the biosynthesized AuNPs using an aqueous extract. The morphological characteristics of AuNPs are spherical with an average size of 127 nm, characterized by various techniques, such as UV-Vis spectroscopy, FT-IR, scanning electron microscopy (SEM) and transmission electron microscopy (TEM). In addition, they also suggested the promising role of biosynthesized AuNPs with enhanced activity against cell proliferation. Finally, they concluded that the *D. pleiantha* rhizome has antimetastatic potential by interfering with the microtubule polymerization in the human fibrosarcoma cell line HT-1080 [132]. It is clear from the previous research that green synthesized AuNPs holds better choice over other methods because of their cost-effectiveness, non-toxicity and feasibility in cancer therapy. However, this theory is backed up by the evidence in Virmani et al., from which the author concludes that biosynthesized nanoparticles have potential antitumor activity compared to chemically synthesized nanoparticles. They reviewed available methods that could be used to predict anticancer activity against many cancerous (HeLa, MCF-7, A549 and H1299) and normal (HEK293) cell lines. The extract derived from *Ocimum tenuiflorum* in the cell viability assay illustrates that biosynthesized AuNPs at lower concentrations were more pronounced and non-toxic, compared to HEK293, which can effectively inhibit the growth of various cancer cell lines with an IC_50_ value of 200 μg/mL. In contrast, the analysis shows that chemically synthesized nanoparticles have contributed negatively to anti-cancer properties even at high concentrations. The results conclude that the use of the chemically synthesized nanoparticles method for cancer therapy offers no obvious advantage [133]. Researchers have reported in their study that the internalization of AuNPs using the extract of *Allium cepa* was non-toxic to cells. *Allium cepa* has various pharmacological properties, including anti-cancer activity; however, over the past year, *Allium cepa*-derived nanoparticles have been of utmost importance in healthcare [134]. Moreover, some important applications of green synthesized AuNPs as an anti-cancer agent are summarized in Table 1. Mostly, the chemically synthesized gold nanoparticles (AuNPs) have been extensively exploited to date; only a few studies have been reported for plant-based green synthesized AuNP in vivo therapy, toxicity, and biodistribution. A study reported that green synthesized gold nanoparticles using leaf extract of *Peltophorum pterocarpum* (*PP*) for doxorubicin delivery both in vitro and in vivo in the C57BL6/J female mice. Administration of biosynthesized doxorubicin-loaded (b-Au-PP-Dox) drug delivery system displayed the significant inhibition of cancer cell growth (A549, B16F10) in vitro as well as inhibition of tumour growth in vivo model compared to free doxorubicin and untreated one [135]. Similarly, leaf extracts of *Mentha piperita*-generated AuNPs were tested against MDA-MB-231 and A549, and normal 3T3-L1 cell lines in vitro, as well as the anti-inflammatory and analgesic activities, were studied on a Wistar rat model. AuNPs showed significant anticancer activities in vitro. However, the in vivo analysis gave positive results for both the activities with less potency as compared to the standard drugs, which suggests that AuNPs might be used in combination with standard drugs to enhance their efficacy [136]. These aforementioned novel in vivo studies have set a new frontier for the potential use of plant-based AuNPs for therapy and drug delivery systems as a cost-effective and eco-friendly approach in the near future. Multifunctionality is the key factor of nanovectors in cancer-specific therapy. Combinatorial therapy with phytoconstituents in cancer therapy has been thoroughly investigated and well documented in the present scenario. More recently, a re-evaluation of this concept has led to the use of a combination of phytochemicals which have been under constant investigation and are particularly used as potent natural anti-cancer agents. This was introduced to overcome some inherent limitations on toxicity, specificity, hazardous and reduced action. The anticancer activity on various cancer cell lines by AuNPs synthesized by using plant extracts are depicted in Table 3.

## 4. Anticancer Mechanism of Green Synthesized Gold Nanoparticles

**Figure 4:**
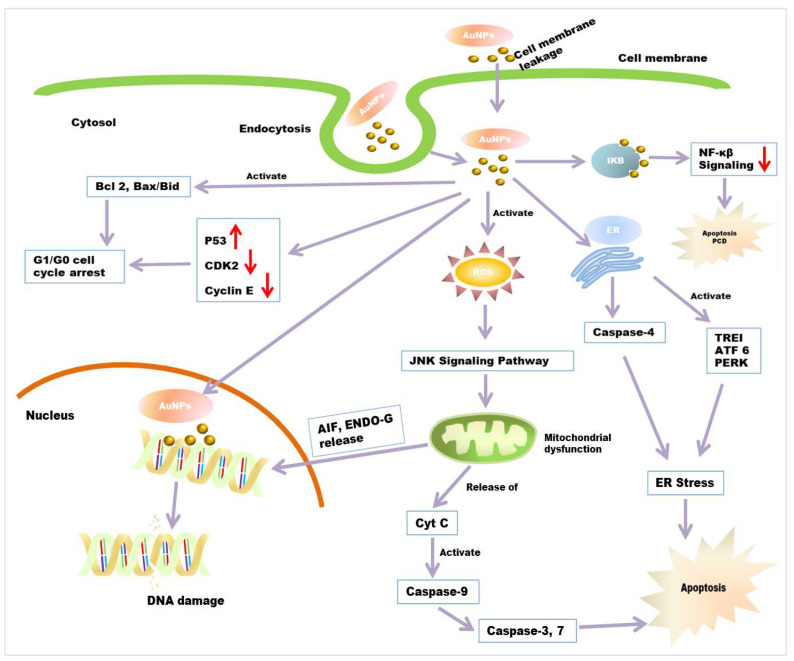
Anticancer mechanism of AuNPs. Entry of AuNPs into the cell’s endocytosis or cellular internalization through a lipid bilayer. AuNPs induce the apoptosis of cancerous cells through ROS-mediated mitochondrial dysfunction and caspase activation. AuNPs causes ER stress through the activation of caspase 4 and stress-related proteins, which also results in apoptosis. Damaged mitochondria release AIF, ENDO-G into the nucleus, which causes the final execution of cell death. AuNPs increases the expression of P53 and decreases the expression of CDK2, Cyclin E. It also elevates the expression of apoptotic gene bid, bax/bcl2, which ultimately leads to G0/G1 phase cell-cycle arrest. AuNP induces PCD and apoptosis through the interfering NF-κB signalling pathway.

There are many anticancer mechanisms of green synthesized nanoparticles that have been reported. However, three proposed mechanisms are well accepted (Figure 4). Firstly, the interaction of NPs with cell membranes interferes with cell permeability and causes mitochondrial dysfunction [185,186]. Secondly, the ROS-induced apoptotic pathway involves an elevated level of ROS and results in oxidative stress and the fragmentation of DNA in the cancerous cell [187]. Thirdly, it can cause interference with the chemistry of proteins/DNA. AuNPs has been reported to be a novel anticancer agent in cancer therapy. It shows size-dependent cytotoxic activity against different cancer cells [188,189]. The use of AuNP in cancer therapy shows minimum side effects and cytotoxic effects to normal (noncancerous) cells [167]. The anticancer mechanism of AuNP is provisionally described in many reports. According to some reports, AuNPs interact with cells in numerous ways; many researchers have reported the cellular internalization of AuNPs [190,191]. The surface properties of AuNPs act as essential factors in cellular internalization. The mechanism behind AuNPs uptake and internalization by cells is due to opposite charges between AuNPs and cell membrane. AuNPs carry positive charges, while cancer/normal cell membranes contain lipids (especially phosphate groups) which carry a negative charge (especially phosphate groups) [190,191]. Additionally, some reports suggested that gold nanoparticles enter into cells through endocytosis. Researchers have reported that the endocytosis of tiny AuNPs and showed aggregation inside HeLa cells [189]. After gaining entry into the mitochondria, they involve different pathways, including impairment of the electron transport chain, structural damage, activation of NADPH enzymes, and the depolarization of mitochondrial membranes [192,193]. AuNPs have been reported to show cytotoxic activity through ROS production [194]. AuNPs cause elevated levels of intracellular ROS in cancerous cells. It was suggested that ROS production is an essential factor in the molecular mechanisms behind the anticancer effect of AuNPs. Excessive ROS production results in irreversible oxidative damage, DNA destruction, and cell death via apoptosis, autophagy, or necroptosis pathways [195]. Apoptosis and autophagy, programmed cell death (PCD) processes, are the major cellular, molecular mechanisms regulating cancer development and progression. Thus, they can be used as important targets for anticancer mechanisms [196]. Increased levels of ROS that are higher than the beneficial physiological concentration causes elevated NADPH level. This can lead to disruption of oxidative balance and impairment of anti-oxidative molecules, resulting in oxidative stress and mitochondrial dysfunction in cancerous cells. Excessive oxidative stress leads to apoptotic cell death. AuNP also causes mitochondrial dysfunction and caspase-dependent apoptosis [194]. Elevated ROS generation also causes depletion of intracellular glutathione. The decline in cellular glutathione concentration and dysregulation of the mitochondrial transmembrane potential are important events occurring early in apoptosis. Mitochondria are the prominent target getting damaged during ROS-induced apoptosis [197]. Due to oxidative stress and membrane permeabilization, mitochondria release some death molecules, such as cytochrome-c, Apoptosis-inducing factor (AIF), Endonuclease G (ENDO-G), etc., from mitochondria to cytosol and nucleus [198,199]. This death molecule causes the final execution of cell death. Caspase-dependent apoptosis is also another leading anticancer mechanism of AuNPs. Caspase zymogens play a crucial role in a biochemical event during apoptosis. The JNK signalling pathway has been reported to be involved in AuNPs-mediated caspase activation and apoptosis process. C-Jun N-terminal kinase (JNK) is an important member of the mitogen-activated protein kinase superfamily, the pathway of which is activated due to elevated levels of ROS [200,201]. Death molecule cytochrome-c release from mitochondria form an apoptosome complex in the cytosol, which results in the activation of initiator caspase-9 [202]. Activated initiator caspases further cleave and activate executioner caspases, such as casp-3, 9, and 7, which can cause cell death. However, mitochondria also release AIF and Endo-G in the cell nucleus, which causes caspase-independent apoptosis [198,203]. Scholars have reported the activation of casp-3 protein in NPs exposed to MDA-MB-231, HeLa, and HCT116 cancer cells, whereas it was absent in untreated cancer cells [204]. They also reported the cleavage of PARP (Poly ADP-ribose polymerase) in the cells exposed with NPs, which is a major downstream substrate for activated casp-3 protease in cancer cells [205]. AuNPs are also found to be responsible for ER stress by activating stress-related proteins, also called ER sensors, IRE1 (inositol-requiring protein-1), ATF-6 (activating transcription factor-6) and PERK (protein kinase RNA (PKR)-like ER kinase in the endoplasmic reticulum. It is reported that AuNP activates caspase 4, which is normally associated with ER stress, resulting in caspase-mediated apoptosis [206]. Researchers have reported that AuNPs induce the elevated expression of the apoptotic gene bid, bax/bcl2 in HCT-116 cells [207]. These lead to cell cycle arrest in the G0/G1 phase and, ultimately, apoptosis. AuNPs has been reported to interfere with and decrease the metabolic activity of cancer cells. They have also been shown to decrease DNA biosynthesis by directly binding to DNA [160] and inhibit DNA repair-related proteins’ expression. According to some reports, AuNP interacts with intracellular components of the NF-κB signalling pathway. NF-B is a key regulator of programmed cell death and is associated with cancer progression by the transcriptional regulation of responsive genes. NF-κB signal transduction proteins IκB kinases (IKK) have cysteine residues and thiol groups, which is the binding substrate of Au-NPs [208,209]. After binding, AuNPs induced apoptosis with dose-dependent decreases in NF-B transcriptional activities.

### 4.1. Applications and Limitations of AuNPs in Drug Delivery for Cancer Therapy

Drug delivery systems are engineered technologies designed for the targeted, efficient delivery of therapeutic agents in a controlled manner. Biomedical engineering contributed to our understanding of the physiological barrier for the drug. This constitutes a step in the right direction in cancer treatment, where the drug has to reach the required location in the desired concentration and remain there for a sufficient period. Most conventional approaches targeting the cancer cells lack adequate contact time, are less stable, lack specificity, and are susceptible to biochemical degradation. Thus, the drug delivery system, based on nanotechnology, is an answer to effective, targeted drug therapy for cancer diagnostics and therapy. A variety of nanotechnological approaches, such as nanoparticles [210], nanoemulsion [211], liposomes [212], niosomes [213], cubosomes [214], spanlastics [215], nanomicelles [216], and nanostructured lipid-carriers (NLC) [217] were used for the drug delivery system used. However, green synthesized metal nanoparticles are an effective strategy and a topic of pivotal importance in the application of drug delivery in therapeutic anticancer research. In recent years, this topic constitutes a new domain with largely unstudied potential. In general, metal nanoparticles, particularly AuNPs, have been observed to be used in various applications, such as drug delivery, molecular imaging and cancer diagnostics and therapy, which depend on the exploitation of important sources, mainly microbes, plants and fruit waste [218]. With advances in the development of nanotechnology-mediated drug delivery systems, plant extracts derived from AuNPs are often selected as having a potential advantage in cancer therapy compared to conventional methods. This has significant advantages in toxicity, eco-friendliness, simplicity and safety, but is not limited to these [79,219]. In addition, the multifunctionality of AuNPs has garnered worldwide attention in the past decade due to the desired particle size, high surface-to-volume ratio, ease of synthesis and high drug loading capacity, making them particularly attractive candidates for drug delivery in cancer therapy. There have been a number of studies that have investigated AuNPs as a promising agent for delivering drugs to tumour sites as either active or passive targeting. There is a growing area of research that uses nanotechnology approaches to prevent ocular cancer. A good overview of earlier work in this area is provided by scholars who suggest that AuNPs significantly suppress VEGF-induced angiogenesis for the retinal neovascularization of endothelial cells and the autophosphorylation to suppress VEGF-2 for the regulation of protein kinases [220]. In one study, it was observed that AuNPs were directly conjugated with the methotrexate and act as a potent anti-cancer agent by disrupting the folate metabolism in the malignant lung cancer cell. Furthermore, the insight into the cancer cell shows that conjugated methotrexate has a higher accumulation in the cell compared to methotrexate alone [221]. This concept has led to a further improvement in cancer diagnostics, with Bhattacharya and colleagues finding that cancer cells that express the folate receptors become more susceptible to targeting AuNPs conjugated with folic acid and PEG-amines [222]. One of the interesting properties of AuNPs is the ‘plasmon resonance’, which is demonstrated in the presence of light (UV and visible) and used to release drugs at the target site, which has been widely emphasized in the literature for disease management. Light-mediated drug delivery is one of the most powerful strategies for in vitro and in vivo analysis in cancer diagnostics. Some time ago, the authors of [223,224] pointed out that the photothermal ablation and photodynamic therapy of AuNPs were also tested for the targeted drug delivery into cancer cells. One particularly notable study describing this fact can be seen in the results of Agasti and co-workers. They showed that illumination with light causes the discharge of the therapeutic anticancer drug 5-fluorouracil from the nanoconjugates of the AuNPs at the target site. These properties can aid in making the drug delivery system more efficient, as more drugs can be efficiently delivered in the light-mediated process compared to the absence of light [225]. Similarly, researchers suggested that DNA-wrapped AuNPs loaded with DOX (GNR@DOX) should be used to treat metastatic breast cancer through chemotherapy and photothermal ablation [226]. Several notable attempts have been made to improve targeted drug delivery, solubility, performance and regulated drug release at specific sites using AuNPs as drug carriers. The pH-mediated drug transport at the respective site is the most appropriate approach, which involves the cleavage of the bond, thus releasing the drug from the metal nanocarrier in an acidic environment, followed by morphological changes in the carrier nanostructure. It has been stated that, stimuli-responsive drug carriers based on AuNPs would be an ideal method of choice, since a lower pH value near the cancer site is effectively exploited for the controlled release of therapeutic drugs from nanoconjugates of AuNPs [227,228]. In this regard, Joshi and group are trying to explore whether or not the chloroquine-AuNPs conjugates have an anti-cancer effect in breast cancer cell lines. They also found that an acidic pH condition near human breast cancer or within the cell provides appropriate conditions for chloroquine to be released from the conjugates. The cytotoxicity of this chloroquine-AuNPs conjugates was quantitatively estimated at different concentrations in MCF-7, which led to an IC_50_ value of 30 ± 5 g/mL. This result demonstrates a better performance of the conjugates in the delivery of chloroquine as well as an improved anti-cancer potential through activation of cell-death mediated by autophagy [229].

In a remarkable study, a new and promising approach using microRNA (miRNA) led to cancer therapy development. In this approach, the authors discussed the miRNAs functionalized nanoconjugates of AuNPs, targeting intracellular proteins by interacting with their transcribed RNA. They inhibited cancer cell viability by minimizing the protein expression by using the targeted miRNA-miR-205 conjugated to AuNPs compared to non-targeted AuNPs [230]. The use of green synthesized AuNPs has recently been of great importance due to their fabrication, monodispersity, ease of synthesis, low toxicity, eco-friendly and useful tools for drug delivery. AuNPs captured more attention due to their excellent properties, such as antibacterial, antifungal, anti-inflammatory and anti-cancer properties, which could pave the way for the development of potential therapeutics. However, there is still a great deal of work to be done in this area.

### 4.2. Gold Nanoparticles in Cancer Cell Diagnosis

**Figure 5:**
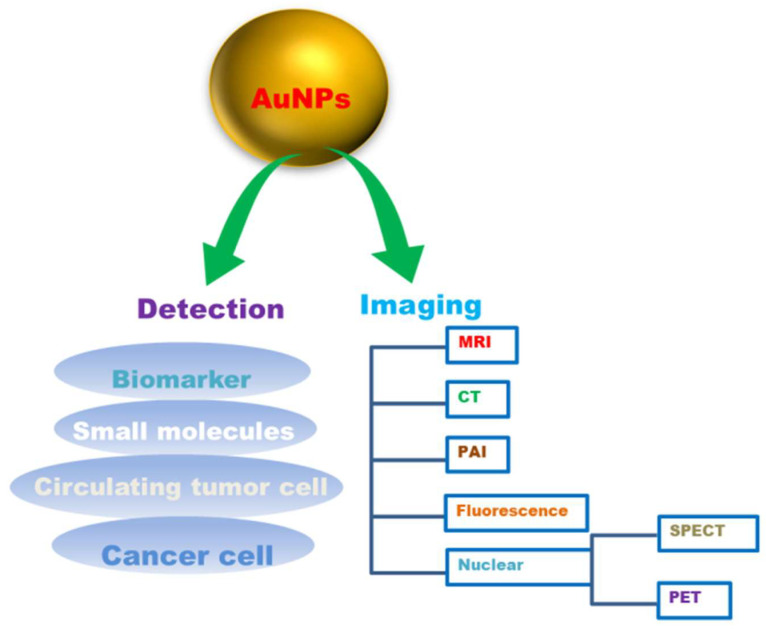
Gold nanoparticles (AuNPs) in cancer diagnosis.

Gold nanoparticles (AuNPs) have attracted a lot of interest in cancer detection and diagnostics because of their intrinsic properties [231]. AuNPs have lower systemic cytotoxicity, are extremely stable, and are nonimmunogenic in vivo. Various targeting strategies, viz., passive targeting or active targeting, can be used to increase the effectiveness of a drug as AuNPs accumulate preferentially in tumours can improve imaging sensitivity [232]. AuNPs have been considered a possible tool for cancer diagnostics and drug delivery due to their unique features. These characteristic features include a high surface area to volume ratio, surface plasmon resonance (SPR), multi-functionalization, easy synthesis, and stable nature. Furthermore, gold nanoparticles’ non-toxic and non-immunogenic nature and their high permeability and retention provide better penetration and accumulation at tumour locations **[233]**. Gold nanoparticles are being used in a variety of novel ways in cancer diagnostics (Figure 5). Gold nanoparticles with unique properties such as smaller size, biocompatibility, and higher atomic number exhibit the potential to conjugate with targeting agents display the potentiality as contrast agents. The mass attenuation of gold at energies > 80 kV was revealed to be more than that of iodine, which also signifies a better contrast agent for imaging [234]. The gold nanoparticles conjugated with several biologically active components, such as amine and thiol groups, may aid biomedical applications, including diagnostics, targeted delivery, imaging, and sensing for electron microscopy markers [235]. Upon binding AuNPs with moieties, the physicochemical properties, such as conductivity, redox behaviour, and SPR, are altered and generate signals that enable the potential of AuNPs as diagnostic agents [236].

AuNPs are being utilized as promising tools for real-time, convenient, and cost-effective cancer diagnosis and detection [237]. The main constraint of various in vitro diagnostic systems is their average detection sensitivity. For example, prostate cancer biomarker detection with enzyme-linked immunosorbent assay (ELISA) can detect a threshold limit of about 0.1 ng/mL, which is normally higher than the concentrations of a cancer biomarker in most serum samples. AuNPs, tend to have high sensitivity for the detection of biomarkers [238]. Their unique physical and optical properties, viz., localized surface plasmon resonance (LSPR), fluorescence resonance energy transfer (FRET), surface-enhanced Raman scattering (SERS), nonlinear optical properties, and quantized charging effect. Enhance AuNPs to sense or detect various targets [238]. AuNPs can help in vitro detection of biomolecules and be used as a diagnostic agent for cancer diagnosis by conjugation with biomarkers viz., oligonucleotides or antibodies to detect the target components [236]. It was reported that it was possible to identify and track the primary glioma cells using mercaptoundecanoic acid-coated AuNPs in mouse brains [239]. Gold nanoparticles (AuNPs) were also reported to be capable of diagnosing with CTC detection. It has been demonstrated that AuNPs can boost the sensitivity and the specificity of CTC detection appliances, and have the ability to aid in cancer diagnosis as well as prognosis [3]. Various imaging technologies, such as computed tomography, ultrasound and magnetic resonance, can provide precise information about disease diagnosis and therapy. AuNPs with unique tuneable chemical and physical properties could thus be an ideal contrast agent for imaging. The use of gold nanoparticles as contrast agents for imaging diagnosis is suitable for various imaging techniques ranging from magnetic resonance imaging (MRI), computed tomography (CT), nuclear imaging, fluorescence imaging, photoacoustic imaging (PAI) to various other imaging techniques (Table 4).

## 5. Phyto-Based Gold Nanoparticles (AuNPs) in Cancer Imaging

Plant extract-synthesized gold nanoparticles have emerged as a promising option for biosensors, immunoassays, and imaging. Interestingly, several types of gold nanoparticles, such as gold nanorods, nanocages, nanostars, nanocubes, and nanospheres, have proven to be efficient tools in cancer research. Their excellent optical and physical properties aided in cancer diagnostics and treatment. Some of the examples using imaging modalities in cancer diagnosis are briefly discussed.

### 5.1. Magnetic Resonance Imaging (MRI)

Magnetic resonance imaging (MRI) is one of the non-invasive diagnostic modalities widely used in the clinic for disease diagnosis, imaging and cell tracking. MRI follows the principle of nuclear spin, i.e., nuclear magnetic resonance (NMR) and proton spin relaxation that aligns parallel or antiparallel to an applied magnetic field [232,253]. Superparamagnetic iron oxide nanoparticles (SPION) were extensively used in MRI as contrast agents, but due to the remarkable toxicity caused by SPION in vivo with ROS generated toxicity, cellular damage (DNA, protein) and inflammation ensued [253,254]. Therefore, SPION is no longer used in MRI clinical applications [255]. Interestingly, AuNPs have no toxic effect and have been extensively utilized for in vivo applications [253]. For instance, grape seed synthesized magnetite-gold nanohybrids (Fe(_3_)O(_4_)/Au), used as contrast agents, were found to be suitable for MRI and CT imaging. The magnetite enables superparamagnetism in MRI, and the gold nanoparticle in the hybrid provides X-ray contrast in CT imaging. The nanohybrids were biocompatible and used for labelling and imaging stem cells [256].

### 5.2. Computed Tomography (CT)

Computed tomography (CT) is one of the most widely employed imaging modalities in clinics based on X-rays. CT is a non-invasive imaging tool that can visualize three-dimensional anatomical images for cancer diagnosis and therapy. AuNPs are recognized as potential CT contrast agents due to their high atomic number and density, which exhibit a high intrinsic X-ray absorption coefficient [257]. Previous studies reported that iodine-based contrast agents showed faster renal clearance and renal toxicity than AuNPs. AuNPs showed 2.7 times more X-ray mass attenuation than iodine, which attracts more attention towards enhancing CT imaging [232,258]. Studies reported that plants that originated from *Hubertia ambavilla* and *Hypericum lanceolatum*, based on shell-like hybrid flavonoid-gold nanoparticles (NPs) hybrid complexes (of about 15 nm, or flower-shaped 40 nm diameter), were developed for a double nanotheranostic activity. This double nanotheranostics implies plasmonic phototherapeutic for cancer therapy by plasmonic phototherapy and X-ray-based computed tomography for visualization by computed tomography [247]. In another study, barley leaf-mediated gold nanoparticles (BL-AuNPs) were synthesized and visualized by CT images in vitro and showed that the BL-AuNPs have better X-ray attenuation ability than the commonly utilized iodinated contrast agent. The study also revealed that BL-AuNPs are a good contrast agent for successful CT imaging of the zebrafish model. These results demonstrated the effective use of BL-AuNPs as a CT imaging contrast agent for disease diagnosis [252].

### 5.3. Fluorescence Imaging

Fluorescence-based optical imaging has great potential for studying biological events at the molecular level and early-stage cancer diagnosis [259]. Fluorescence imaging relies on a linear relationship between the intensities of fluorescent signals emitted by the excitation of fluorescent components and the amount of fluorescent material at a certain range [253]. In a study, *Olax scandens* leaf extract synthesized fluorescently labelled AuNPs (AuNPs-OX) were added to lung (A549), breast (MCF-7) and colon (COLO 205) cancer cells and were found to be helpful for the detection of the cancer cells. AuNPs-OX displayed strong red fluorescence in cancer cells compared to aqueous leaf extract alone [260].

### 5.4. Photoacoustic Imaging

Photoacoustic imaging (PAI) is a real-time biomedical imaging modality with non-invasive properties, which provides functional details about tissues’ cellular structure and molecular events, by applying endogenous and exogenous contrast agents [261]. The PAI technique relies on energy conversion from light to sound [262]. Imaging agents with high photothermal conversion potential are a prerequisite for better photoacoustic contrast [263]. Gold nanoparticles as contrast agents have tremendous potential for PAI due to their intrinsic and geometrically induced optical properties. For example, Cinnamon-synthesized AuNPs provided a suitable diagnostic agent for in vitro and in vivo imaging. These AuNPs are biocompatible and pure, so can be utilized for in vivo applications. The uptake Cinnamon-AuNPs were visualized in PC-3 and MCF-7 cells in vitro and detected by photoacoustic signals. Moreover, biodistribution studies in normal mice showed that these AuNPs accumulated in the lungs, which further indicated the use of AuNPs as contrast agents for targeting [264].

### 5.5. Application of AuNPs in Magnetic Particle Imaging (MPI)

The concept of magnetic particle imaging (MPI) was introduced by researchers, and relies on the non-linearity of the magnetization curves of ferromagnetic materials. With this discovery, it becomes possible to obtain high-resolution images of magnetic tracers used for diagnosis. They have also demonstrated the feasibility of the imaging method through the achievement of the image with resolution below 1 mm [265]. MPI, as a tracer-based tomographic imaging modality, can determine the spatial distribution of magnetic nanoparticles (MNPs) and has applications in vast biomedical arenas, such as cell targeting, drug delivery, and diagnostic imaging, as well as in magnetic hyperthermia [266]. Nowadays, magnetic particle spectroscopy (MPS) or magnetisation response spectroscopy (MRS) is evolving as a versatile measurement tool derived from MPI. This is basically designed to characterised superparamagnetic iron oxide nanoparticles (SPIONs) regarding their applicability in MPI [267]. Most of the recent works have focused on the application of iron-oxide-based magnetic nanoparticles (MNPs) in MPI. However, AuNPs in combination with MNPs also find their applicability in MPIs due to their various physicochemical properties. A nanoparticle possessing both plasmonic as well as magnetic properties within the same nanosystem has efficient application in magnetic particle imaging, along with other image-guided therapy. The core shell-shaped gold nanoparticles coated with polyethylene glycol, having a magnetic cum plasmonic effect MNPs@Au, was synthesised by the reduction of HAuCl_4_ having a potential application in MPI [268]. The star-shaped plasmonic shell of gold nanobranches with high aspect ratio, with a superparamagnetic iron core offering a unique nanostar structure that is useful for the application in MPI, is proposed by a group of researchers. It was found that the model drug molecules can bind to the core-shell Nanostars and were released when illuminated with near infrared (NIR) due to heat release from the core shell Nanostars [269].

## 6. Immunomodulatory Properties of AuNPs

Gold nanoparticles (AuNPs) have been shown to have several immunomodulatory properties. Dendritic Cells have been considered an important model to investigate potential immunomodulatory properties of NPs [270]. According to some reports, in the absence of pro-inflammatory stimuli (IL-1β, TNF-α), AuNPs do not have any immunomodulatory properties and can be considered immunologically safe. However, under pro-inflammatory conditions, 26 nm AuNPs may show immunomodulatory capacities [271]. AuNPs also inhibit dendritic cell (DC) activation, reduced expression of activation marker CD86, decrease in the secretion of pro-inflammatory cytokines IL-12 and IL-27, and concomitant upregulation of the anti-inflammatory receptor ILT3, which ultimately results in anti-inflammatory responses. Moreover, several reports suggested other different anti-inflammatory responses of AuNPs [137,272]. Pro-inflammatory cytokines IL-1β, TNF-α and IFN-γ, produced by innate immune component macrophages and natural killer (NK) cells, were generally released in response to bacterial and tissue infections. However, prolonged inflammation can contribute to the etiology of several diseases, such as rheumatoid arthritis, inflammatory bowel disease, multiple sclerosis, psoriasis, and eczema [273]. The expressions of these cytokines, including IL-1β, IL-6 and TNF-α, were altered in rats and animals after being injected with AuNPs [274]. Citrate-stabilized AuNPs downregulate the cellular response induced by IL-1β both in vivo and in vitro, resulting in anti-inflammatory responses. It has been signified that the increased production of reactive oxygen species (ROS) in response to AuNPs also involves anti-inflammatory actions [275]. Some studies have suggested that (Mangiferin) MGF-AuNPs effectively target tumour-associated macrophages (TAMs), which show immunomodulatory properties by infiltrating most solid tumours. Macrophages have pro-tumour or anti-tumour phenotypes. Classically activated macrophages (referred to as M1), and alternatively activated macrophages (referred to as M2), fit two extremes within the spectrum of the macrophage phenotypes [276]. M1 macrophages are involved in pathogens phagocytosis and the activation of antitumor activity by IL-12–dependent natural killer (NK) cell recruitment. However, tumour-associated macrophages (TAM) exhibit the expression of the M2-like phenotype along with high IL-10, high arginase-1 and low IL-12 expression, which is involved in pro-tumorigenic activities and ultimately contributes to drug resistance to several diseases. MGF-AuNPs initiate macrophage re-education from pro-tumour M2 macrophages to the antitumor M1 phenotype, resulting in tumour growth restriction and metastasis by eliminating cancer cells [277,278].

## 7. Limitations of Using AuNPs in Biomedical Applications

Toxicity is considered a major concern regarding the limitations of using AuNPs in biomedical applications. Different properties of AuNPs, including shape, size, surface chemistry, targeting ligand, elasticity, and composition, influence their toxicity to biological systems [279]. Previous research investigations have shown that chemically synthesized AuNPs that are smaller than 20 nm are highly toxic to stem cells, affecting cellular DNA methylation and hydromethylation patterns, whereas green synthesized AuNPs have been proven to be an effective anticancer drug carrier, delivering drugs to cancer cells and killing them. The toxicity of AuNPs is thus dependent on the synthesis method, which determines their size, morphology, topology, and surface functional group [280]. Moreover, in combination with the complexity and heterogeneity among human cells and tissues, it is challenging to comprehensively probe the effect and response of the biological system to the administration of AuNPs. In a study conducted by Khan and colleagues, mice were given different types of dendrimers-encapsulated AuNPs (5–22 nm) with positive, negative, or neutral surface charges. The gold content of the various organs, blood, and excrements was determined after the sacrifice. The researchers came to the conclusion that nanoparticle size and surface charge affect biodistribution. The smallest positive particles concentrating in the kidneys and the bigger ones accumulating in the spleen, liver, lungs, and heart. Even though AuNPs are said to be non-toxic by nature, it is crucial to distinguish between the toxicity of the nanoparticles and the toxicity of the capping ligands. Certain capping ligands may be more toxic than others [281]. Additionally, there is currently no significant standardized assay available that could be applied to test the AuNPs toxicity effect. The lack of standardized assays results in varying interpretations or assumptions. This is also a significant limitation of nanoparticles administration. It is important to emphasize that AuNPs are biodegradable. The NPs might take longer to excrete and they may accumulate in the liver and spleen. The bioaccumulated AuNPs may interfere with different diagnostic techniques, or accumulated AuNPs will exhibit catalytic properties [282]. Therefore, the biodistribution and excretion factors must be studied comprehensively in various animal models with appropriate sample sizes and robust statistical studies. In combination with potential toxicity, all of these concerns are immense limitations of AuNPs on various successful clinical applications. More research is needed on this domain to overcome these limitations. Elimination of accumulating NPs from cancer patients is challenging. No significant technology is currently available to date. However, Shahidi Bonjar described a “Nanogold detoxifying machine” to filter idle AuNPs from the blood of treated cancer patients [283]. The equipment, which resembles a “hemodialysis machine”, might improve the safety of AuNPs therapy for specific tumours and prevent AuNP accumulation in non-target tissues or organs following treatment.

## 8. Future Prospect and Conclusions

In the 21st century, the biogenesis of nanomedicine has a great deal of potential for treating cancer by developing efficient anticancer nanomedicine and drug delivery systems that deliver potent drugs effectively to specific targeted areas. Looking at the enormous significance of AuNPs over the past few years, and the safety and biocompatibility of green synthesis methods, it is envisaged that green synthesized AuNPs will eventually be beneficial in cancer therapy and diagnostic area. Plant-based AuNPs are likely to be highly advantageous in the fight against cancer due to their biocompatibility and pronounced anticancer therapeutic and diagnostic efficacy. These AuNPs may pave the way for developing a new generation of anticancer medicines in this fashion. Because of its pharmaceutical applications, the AuNPs industry has already evolved into a massive economy and is expected to increase more worldwide. If significant innovations and research is performed, plant-based AuNPs will provide a considerable portion of this percentage. Plant-based synthesis can provide a convenient and cost-effective outlet for AuNPs. Extensive research should be focused on designing and engineering the synthesis of plant-based AuNPs to meet the expectations commonly placed on chemically synthesized AuNPs. The green synthesis of AuNPs is still in its early stages. However, the in vivo study of AuNPs in various animals, along with three-dimensional spheroids and organoids models, has received little attention in the published literature. More research on this is needed, which will boost confidence in translational to clinical studies for the effective and safe utilization of AuNPs in cancer patients. The mass-scale manufacture of cost-effective and efficient AuNPs functionalized with moieties such as anticancer drugs and targeting ligands are required to achieve this.

Thus, certain efforts were needed to enhance nanoparticles synthesis at a large scale, successful clinical trial, resulting in nanoparticles with potential therapeutic applications, such as personalized cancer therapy.

## Figures and Tables

**Table 1:** The effect of Cl^−^ ion concentration and reductant on AuNPs size.

HAuCl_4_ (0.25 mM) + 5% Sodium citrate (Both the reactants are in 5:1 ratio)	**Concentration of NaCl (mM)**	**UV-Visible Absorption Maxima [λ_max_ (nm)]**	**Size Ranges from TEM** **(nm)**
	1	517	19 nm (±7)
	5	520	25 nm (±11)
	10	525	38 nm (±21)
	15	528	40 nm (±31)
	20	531	47 nm (±36)
HAuCl_4_ (0.25 mM) + Sodium borohydride (NaBH_4_)	0	490	3
	20	520	12

**Table 2:** Green synthesis of Gold Nanoparticles (AuNPs) from different plants.

Plant	Plant Part	Reactive Compound	Salt Solution	Size (nm)	Shape	Characterization	References
*Artemisia vulgaris* (Mugwort)	Leaves	Polyphenols, flavonoids, terpenoids	HAuCl_4_	50–100	Spherical, triangular, hexagonal	UV-vis Spectroscopy, XRD, FT-IR, DLS, ZP, TEM and EDX.	[84]
*Clitoria ternatea* (Asian pigeonwings)	Leaves	Alcoholic, amine groups, halo compounds	HAuCl_4_	100	Rod	UV-vis spectroscopy, FTIR, XRD, TEM, EDX	[85]
*Murraya koenigii* Spreng (Curry leaves)	Leaves	Polyphenols, quercetin, quercetin-3-glucoside, flavonoids	HAucl_4_	20–40	Spherical	XRD, EDX, FT-IR, HPLC, TEM, UV-vis spectra, Fluorescence microscopy.	[86]
*Artocarpus* *hirsutus* (Wild jack)	Leaves	Polyphenols, flavonoids, terpenoids	HAuCl_4_	5–40	Spherical	XRD, UV-visible spectra, FT-IR and TEM	[87]
*Justicia glauca* (Thaasi murungai)	Leaves	Lignans[(+) pinoresinol, (+)-medioresinol], alkaloids, flavonoids, steroids (sitosterol-3-0-glucoside), terpenoids	HAuCl·3H_2_O	32	Hexagonl, spherical	UV-vis spectral analysis. X-ray, XRD, TEM, FTIR, EDX, CV, DPV.	[88]
*Terminalia arjuna* (Arjun tree)	Leaves	Arjunetin, leucoanthoc-yanidins, hydrolyzable tannins	HAuCl_4_	20–25	Spherical	UV-visible spectra, FT-IR, XRD, AFM and TEM	[89]
*Memecylon umbellatum*	Leaves	Protein, saponins, polyphenols, carbohydrate	HAuCl_4_, AgNO_3_	15–25	Spherical, triangular, hexagonal	UV-visible spectra, FTIR, energy-dispersive x-ray spectroscopy, TEM,	[90]
*Mangifera indica*	Leaves	Terpenoids, flavonoids, thiamine	HAuCl_4_·3H_2_O	17–20	Spherical	UV-vis, TEM and XRD.	[91]
Olive	Leaves	Proteins, oleoropein, apigenin-7-glucoside, luteolin-7-glucoside	HAuCl_4_·3H_2_O	50–100	Triangular, spherical, hexagonal	UV-vis spectroscopy, photoluminescence, TEM, XRD, FTIR and thermogravimetric analysis.	[92]
*Coreopsis* *lanceolate*	Leaves	Antioxidants like sugars, flavonoids	HAuCl_4_,	20–30	Spherical, quasi spherical	UV-vis spectroscopy, TEM and spectro fluorimetry	[93]
*Cassia auriculata* (Matura tea tree)	Leaves	Polysaccharides, flavonoids	AuCl_3_, Au_2_Cl_6_	15–25	Spherical, triangular, hexagonal	X-ray diffraction, TEM, SEM-EDAX, FT-IR and visible absorption spectroscopy.	[94]
*Lonicera Japonica* (Japanese honeysuckle)	Flower	Amino acids	AgNO_3_, HAuCl_4_	8	Triangular tetrahedral	UV-vis spectrophotometer, FTIR, XRD, EDX, SEM and HRTEM.	[95]
*Nyctanthes* *arbortristis* (Night flowering jasmine)	Flower	alkaloids, flavonoids	HAuCl_4_	15–25	Spherical	UV-vis spectro photometer, TEM, XRD, FTIR, NMR.	[96]
*Guazuma* *ulmifolia* (Bay cedar)	Bark	Tannins, proanthocya-nidins, precocene, catechins.	HAuCl_4_·3H_2_O, AgNO_3_	20–25	Spherical	UV-vis spectroscopy, FT-IR, XRD, AFM and HR-TEM analyses	[97]
*Salix* (Willow tree)	Bark	Tannins, alkanoids, flavonoids, alkaloids.	AuCl_4_H_9_O_4_	15	Spherical	UV-vis spectroscopy, XRD, TEM, and HR-TEM.	[98]
*Acacia nilotica* (Gum Arabic tree)	Bark	Protein, phenols, tannins, terpenoids, saponins	HAuCl_4_·3H_2_O	10-15	Unshaped, quasispherical	UV-vis spectroscopy, XRD, EDX, TEM, FTIR, DPV.	[99]
*Musa paradisiaca* (Banana)	Peel	Phenolic compounds, gallocatechin, dopamine	HAuCl_4_	50	Spherical	UV-vis spectroscopy, FTIR, XRD, TEM, Zeta potential analysis and EDX.	[100]
*Mangifera indica* Linn (Mango)	Peel	Phenols, carboxylic acids	HAuCl_4_	3.26–21.68	Quasi-spherical	UV-vis spectrum, XRD, TEM, and FTIR.	[101]
*Terminalia arjuna* (Arjun tree)	Peel	Polyphenols	HAuCl_4_	60	Triangular hexagonal pentagonl	UV spectroscopy, HRTEM, XRD, FESEM, EDX, DLS, and zeta potential analyses.	[102]
*Lantana camara* (Wild sage)	Fruit	Ursolic acid, iridoid glycosides, monoand sesquiterpe-nes, flavonoids	HAuCl_4_	150–300	Triangular	UV-vis-NIR, TEM, SAED, DLS, and XRD techniques.	[103]
Citrus (Lemon, tangerine, orange)	Fruit	Citric acid, proteins	HAuCl_4_	32.3, 43.4, 56.7	Spherical, triangular	UV-visible spectra. TEM XRD, SEAD, DLS.	[104]
*Citrus maxima* (Pomelo)	Fruit	Polypeptides/proteins, terpene, ascorbic acid	HAuCl_4_·4H_2_O	15–35	Spherical	UV-vis spectroscopy, SEM, XRD and FTIR.	[105]
Pear	Fruit	Sugars, amino acids, proteins	HAuCl_4_	20–400	Triangular hexagonalpolyhedral	UV-vis spectroscopy, TEM, AFM, XRD, XPS, EDAX.	[106]
*Sterculia* *acuminate* (Pola plant)	Fruit	Phenolic compounds	HAuCl_4_	9.37–38.12	Spherical	TEM, X-ray diffraction, UV-vis spectroscopy and FTIR, and X-ray photoelectron spectrometry.	[107]
*Pistacia* *integerrima* (Zebra wood)	Galls	Monoterpenes, triterpenoids, sterols, dihydromal—valic acid.	HAuCl_4_·3H_2_O	20–200	Grain-like	UV-vis spectroscopy, FTIR and SEM.	[108]
*Abelmoschus* *esculentus* (Okra)	Seed	Proteins, polysaccharides, glycoprotein	HAuCl_4_	45–75	Spherical, uneven shape	UV-visible spectroscopy, XRD, FTIR, AFM, FESEM and EDX.	[109]
*Theobromo* *cacao* (Cocoa)	Seed	Polyphenols	HAuCl_4_	150–200	Spherical	UV-vis-NIR spectrophotometer, SPR, TEM, XRD, FTIR, XPS	[110]
*Hevea* *brasiliensis* (Para rubber tree)	Latex	isoprene, proteins	AuCl_3_, Au_2_Cl_6_	50	Spherical, triangular	UV-vis spectroscopy, SEM, TG/FT-IR, XED.	[111]
*Zingiber officinale* (Ginger)	Rhizome	Oxalic acid, ascorbic acid, Phenylpropanoids, zingerone.	HAuCl_4_, AgNO_3_	10–20	Spherical, triangular, truncated triangular, hexagonal	UV-visible spectroscopy, SEM-EDS, TEM, XRD, FTIR.	[112]
*Curcuma longa* (Turmeric)	Rhizome	Phenolic (curcumin), triterpenoids, alkaloid, sterols.	HAuCl_4_, AgNO_3_	5–60	Oblong spherical	UV-vis spectroscopy, XRD, TEM, HR-TEM, thermogravimetric analysis.	[113]
*Panax ginseng* C.A. Meyer (Korean red ginseng)	Rhizome	Saponin glycoside (ginsenoside), polysaccha-rides, flavones, peptide glycans	HAuCl_4_·3H_2_O	2–40	Spherical	UV-visible spectra, TEM, FTIR.	[114]
*Acorus calamus* (Sweet flag)	Rhizome	Asarone, caryophyllene, isoasarone, methyl isoeugenol, safrole.	HAuCl_4_·3H_2_O	10	Spherical	UV-visible spectral analysis, XRD and FT-IR, SPR, HR-TEM, SEM with EDAX.	[115,116]
*Acacia nilotica* (Gum Arabic tree)	Bark	Protein, phenols, tannins, terpenoids, Saponins.	HAuCl_4_·3H_2_O	10–50	Unshaped, quasispherical.	UV-vis spectroscopy, XRD, TEM, EDX, DPV and FTIR.	[99]
*Guazuma* *ulmifolia* (Bay cedar)	Bark	Tannins, proanthocya-nidins, precocene, catechins.	HAuCl_4_·3H_2_O and AgNO_3_	20–25	Spherical	UV-vis spectroscopy, FT-IR, XRD, AFM and HR-TEM analyses.	[97]
*Areca catechu* (Pinang palm)	Nut	Polyphenols, fats, proteins, carbohydrate, flavonoids.	HAuCl_4_	13.70	Spherical	UV-visible, TEM, XRD, and FTIR.	[117]
*Momordica* *Cochinchinensis*.	Biomass	Proteins	HAuCl_4_	10–80	Spherical, oval, triangular	UV-visible, FT-IR, XRD, TEM, SPR and EDX	[118]
Palm oil mill effluent	Palm oil	Proteins, flavonoids, reducing sugars, alkaloids	HAuCl_4_·3H_2_O	13–25	Spherical	UV-vis spectroscopy, TEM, XRD, and FTIR.	[119]
*Macrotylomauniflorum* (Horse gram)	Whole plant	Proteins, carbohydrate, antioxidant	HAuCl_4_·3H_2_O	14–17	Spherical	UV-visible spectroscopy, TEM, XRD and FTIR analysis.	[120]

**Table 3:** Showing anticancer activity of gold nanoparticles using plant extracts and characterization technique.

Sl No.	Plant	Extract Used	Anticancer Activity Type	Characterization	Shape	Size	References
1	*Brazilian Red Propolis*	Hydroethanolic extract	Bladder (T24) and prostate (PC-3) cancer cell line	SPR, UV-Vis spectroscopy, NTA, TEM, EDXS, SAED, FTIR, TGA,	Spherical	8–15 nm	[137]
2	*Abies spectabilis*	Aqueous extract	Bladder cancer T24 cell line	TEM, SAED, UV-visible spectroscopy, EDX, FTIR, AFM, XRD	Spherical	20–200 nm	[138]
3	*Benincasa hispida*	Aqueous extract	HeLa cells and normal osteoblasts cell line	UV-Visible Spectroscopy, DLS, Zeta sizer, TEM, FTIR	Spherical	22.18 ± 2 nm	[29]
4	*Butea monosperma*	Aqueous water extract	Normal endothelial cells (HUVEC, ECV 304) and cancer cell lines (B16F10, MCF-7, HNGC2 and A549)	UV-visible spectroscopy, XRD, TEM, FTIR, DLS, XPS	Spherical, rod, triangular, hexagonal	30 nm	[139]
5	*orchid*	Orchid plant extract(whole)	Breast cancer AMJ 13 cell lines	UV-Vis spectroscopy, TEM, AFM, FTIR	Spherical	14–50 nm	[140]
6	*Taxus baccata*	Ethanolic extract	Breast (MCF7), cervical (HeLa), ovarian (Caov-4) cancer cell line	UV-Vis spectroscopy, TEM, Zetasizer, FTIR, EDX, AFM	Spherical, semispherical, hexagonal, triangular	20 nm	[37]
7	*Marsdenia tenacissima*	Leaf extract	A549 lung cell line	UV-vis, spectroscopy, AFM, EDS, TEM, FTIR, XRD, SAED	Spherical, anisotropic	50 nm	[141]
8	*Argemone mexicana* *L.*	Aqueous extract	Human colon cancer cell line, HCT-15	TEM, XRD, FTIR	Hexagonal	20–40 nm	[142]
9	*Couroupita guianensis*	Aqueous extract	Leukemia cell line	UV-vis spectroscopy, FTIR, XRD, SEM, TEM	Spherical, triangular, tetragonal, pentagonal	7–48 nm	[143]
10	*Lycium chinense*	Fruit extract	Human breast cancer MCF7 cell line and non-diseased RAW264.7 (murine macrophage) cells	UV-vis spectroscopy, FTIR, XRD, FETEM, EDX, SAED	Poydispersed, agglomerated	20–100 nm	[144]
11	*Tabebuia argentiea*	Aqueous, flower extracts	Hepatic cells (Hep G2) cell line	EDX, SEM	Spherical	56 nm	[145]
12	*Dendropanax morbifera*	Aqueous, leaf extract	A549 lung cancer cell line and human keratinocyte cell line	UV-Vis spectroscopy, EDX, FETEM, XRD, DLS	Polygonal, hexagonal	5–10 nm	[146]
13	*Halymenia dilatata*	Aqueous extract	Human colorectal adenocarcinoma cells (HT-29)	UV-Vis spectrophotometry, FTIR, XRD, FESEM, HRTEM, EDX, Zetasizer, DLS	Triangular, spherical	16 nm	[147]
14	*Dracocephalum kotschyi*	Leaf extract	Cervical cancer (HeLa), leukemia (K562) cell lines	UV-Vis spectrophotometry, TEM-SAED, SEM-EDAX, XRD, Zeta potential, DLS, FTIR	Spherical	11 nm	[148]
15	*Sargassum glaucescens*	Water extract (seaweed)	Cervical (HeLa), liver (HepG2), breast (MDA-MB-231), leukemia (CEM-ss) cell lines	UV-Vis spectroscopy, SEM, TEM, EDX	Spherical	3.65 ± 1.69 nm	[149]
16	*Trachyspermum ammi*	Seed extract	HepG2 cancer cell line	UV-Vis spectroscopy, XRD, TEM, DLS, FTIR	Spherical	16.63 nm	[34]
17	*Musa acuminata colla*	Aqueous, Flower extract	MCF-7, normal Vero cell line	UV-Vis, FTIR, XRD, SEM, EDAX	Spherical	10.1–15.6 nm	[150]
18	*aegle marmelos, eugenia jambolana and soursop*	Fruit extract	Human breast cancer cell line MCF-7	UV-Vis spectroscopy, TEM, FTIR, Zeta potentiometer	Spherical	18.28,16 nm	[151]
19	*Muntingia calabura*	Aqueous, fruit extract	Hep2 cells line	UV-Visible spectroscopy, DLS, FTIR, TEM	Spherical, oval	27 nm	[152]
20	*Nigella sativa*	Ethanolic leaf extract	Hep-G2 liver cancer cell line	TEM, XRD, EDS, FTIR, UV-Vis spectroscopy	Anisotropic	13–78 nm	[153]
21	*Marsilea quadrifolia L.*	Aqueous Leaf extract	PA-1 and A549 cell line	TEM, XRD, EDX, FTIR, UV-Vis spectroscopy	Spherical	10–40 nm	[154]
22	*Ocimum sanctum*	leaf extract	Dalton’s lymphoma	UV-Vis spectroscopy, XRD, SEM, TEM, FTIR	Spherical	12–20 nm	[155]
23	*Bauhinia tomentosa Linn*	Aqueous, leaf extract	A549, HEp-2, MCF-7 cell line	FESEM, HRTEM, FTIR, EDX, XRD, TGA, UV-Vis spectroscopy	Spherical	11.5–40 nm	[156]
24	*Shorea tumbuggaia*	Bark extract	Thyroid cancer (SW579) cell lines	XRD, HRTEM, SAED, DLS, zeta potential, FTIR, UV-Vis spectroscopy	Spherical	20 nm	[157]
25	*walnut green*	Shell extract	MCF7 cells	UV-Vis spectroscopy, XRD, TEM,	Spherical, triangular	10–50 nm	[158]
26	*Cassia tora*	Leaf extract	Colon cancer cells	UV-Visible spectroscopy, FTIR, TEM, zeta potential, dark field microscopy	Spherical	57 nm	[159]
27	*Abutilon indicum*	Water extract of leaves	HT-29 cells	UV-Vis spectroscopy, SPR, FTIR, DLS, EDAX, TEM, zeta potential, XRD, TGA	Spherical	1–20 nm	[160,161]
28	*Catharanthus roseus*	Water extract of Leaves	MCF7 and HepG2 cell line	UV-Vis spectroscopy, HRTEM, XRD, TEM	Spherical and triangular	15–28 nm	[160,162]
29	*Gymnema sylvestre*	Water extract of leaves	HT29 cell line	UV-Vis spectroscopy, SEM, EDAX, XRD, FTIR	Spherical	72.8 nm	[160,163]
30	*Hibiscus sabdariffa*	Water extract of leaves	U87 cell line	UV-vis spectroscopy, XRD, FTIR, XPS, TEM	Spherical	10–60 nm	[164,165]
31	*Hygrophila spinosa*	Water extract of leaf	HeLa cell line	XRD, SEM, EDAX, DLS, FTIR and UV-Vis spectroscopy.	Triangular and spherical	50–80 nm	[160,166]
32	*Moringa oleifera*	Water extract of leaves	A549 and SNO cells	DLS, TEM, UV-Vis spectroscopy, zeta potential	Spherical and polyhedral	10–20 nm	[160,167]
33	*Podophyllum hexandrum L.*	Water extract of leaves	HeLa cell line	UV-Vis spectroscopy, TEM, XRD, FTIR	Spherical	5–35 nm	[160,168]
34	*Elettaria cardamomum*	Seed, aqueous extract	HeLa cancer cell line	UV-Vis spectrophotometer, SAED, FTIR, XRD	Spherical	15.2 nm	[169]
35	*Coleous forskohlii*	Root extract	HEPG2 liver cancer cell line	UV-Vis spectrophotometer, HRTEM, FTIR, XRD, PSA	Spherical	10–30 nm	[170]
36	*Scutellaria barbata*	Aqueous extract	Pancreatic cancer cell lines (PANC-1)	UV-visible spectroscopy, TEM, SAED, AFM, FTIR, DLS, EDX	Spherical	0.4 μm–1 μm	[171]
37	*Sargassum incisifolium*	Aqueous extract	HT-29, MCF-7 cancer cell line, MCF-12a non cancer cell line	TEM, XRD, UV-Vis spectroscopy, zeta potential, FTIR, EDX, DLS, ICP-AES	Spherical	12.38 nm	[172]
38	*Panax notoginseng*	Leaf extract	PANC-1 cell line	UV-Vis spectroscopy, TEM, DLS, FTIR, AFM, SAED	Hexagonal, spherical, oval, triangular	80–12 nm	[171]
39	*Antigonon leptopus*	Leaf extract	Human adenocarcinoma breast cancer (MCF-7) cells	UV-Vis spectroscopy, XRD, SAED, FTIR, HRTEM, EDX	Spherical	13–28 nm	[173]
40	*Mukia Maderaspatna*	Aqueous, leaf extract	MCF 7 breast cancer cell line	UV-Vis spectroscopy, EDAX, SEM, TEM, FTIR	Spherical, circular, triangular	20–50 nm	[174]
41	*Hevea brasiliensis*	Latex extract	CHO-K1 cell line	UV-Vis spectroscopy, XRD, TEM, FTIR	spherical	9 nm	[175]
42	*Lonicera 4 japonica*	Flower extract	Cervical cancer (HeLa) cell line	UV-Vis spectroscopy, EDX, XRD, GCMS, TEM, FTIR	Polydisperse (spherical, triangular, hexagonal)	10–40 nm	[176]
43	*Anacardium occidentale*	Leaf extract	MCF-7 cell line	UV-Vis spectroscopy, TEM, XRD, FTIR	Spherical	10–30 nm	[177]
44	*Sasa borealis*	Aqueous, leaf extract	AGS (Gastric adenocarcinoma) cell line	UV-Vis spectroscopy, TEM, EDX, XRD, FTIR, GCMS	Oval, spherical	10–30 nm	[178]
45	*Alternanthera Sessilis*	Aqueous, leaf extract	Cervical cancer (HeLa) cell line	UV-Vis spectroscopy, HRTEM, EDX, SAED, AFM, FTIR	Spherical	20–40 nm	[179]
46	*Bauhinia purpurea*	Aqueous, leaf extract	Lung carcinoma cell line (A549)	UV-Vis spectroscopy, HRTEM, SAED, XRD, EDX, FTIR	Spherical, polygonal	20–100 nm	[180]
47	*Crassocephalum rubens*	Aqueous, leaf extract	MCF-7 and Caco-2 cells	UV-Vis spectroscopy, TEM, FTIR	Spherical	20 ± 5 nm	[181]
48	*Backhousia citriodora*	Aqueous, leaf extract	MCF-7 breast cancer cell line and the HepG2 liver cancer cell line	UV-Vis spectroscopy, TEM, zeta potential, XRD, FTIR	Spherical	8.40 ± 0.084 nm	[182]
49	*Petroselinum crispum*	Aqueous, leaf extract	Human cancerous colorectal cell line	UV-Vis spectroscopy, TEM, EDX, FTIR.XRD	Spherical, semi-rod aggregates, flower-shaped nanoparticles	20–80 nm	[183]
50	*Indigofera tinctoria*	Aqueous, leaf extract	lung cancer cell line A549	UV-Vis. spectroscopy, FTIR, XRD, TEM, EDX, AFM	Spherical, triangular, hexagonal	6–29 nm	[184]

**Table 4:** Gold nanostructures used in imaging-based diagnostics.

Nanostructure	Tumour Model/Cell Line	Imaging Modality	References
AuNPs	Prostate tumour (PC3)	MRI	[240]
AuNPs	Prostate tumour (PC3)	CT	[241]
[198Au]AuNCs	-	SPECT	[242]
64CuAuNCs	Breast tumour (4T1)	PET	[243,244]
AuNCs	Breast tumour (MDA-MB-231)	Fluorescence	[245]
AuNPs	A431 cells	PAI	[246]
AuNPs	-	CT	[247]
Au/Ag hybrid nanoparticles	SKOV3	PA	[248]
AuNU-pHLIP	MCF-7	CT/PA	[249]
AuNR-SiO_2_-PFP	A375	US/PA	[250]
DT-AuNR/PDA bowl spadix-bract NP	Hep-G2, HeLa, MCF-7	CT/PA	[251]
BL-Au NPs	Zebrafish model	CT	[252]

## Abbreviations

AFM—Atomic force microscopy, CV—Cyclic voltammetry, DLS—Dynamic Light Scattering, DPV—Differential pulse voltammetry, EDS/EDX—Energy Dispersive X-ray Spectroscopy, EMA—European Medicines Agency, FDA—Food and Drug Administration, FTIR—Fourier transform infrared spectroscopy, GCMS—Gas Chromatography–Mass Spectrometry, HPLC—High-performance liquid chromatography, HRTEM—High-Resolution Transmission Electron Microscopy, ICPAES—Inductively Coupled Plasma Atomic Emission Spectroscopy, PSA—Particle Size Analysis, SPR—Surface Plasmon Resonance, SAED—Selected Area Diffraction, TGA—Thermogravimetric Analysis or Thermal Gravimetric Analysis, NTA—Nanoparticle Tracking Analysis, SAED—Selected area electron diffraction, XRD—X-ray diffraction, XPS—X-ray Photoelectron Spectroscopy, NMR—Nuclear Magnetic Resonance, SEM—Scanning Electron Microscopy, SPR—Surface Plasmon resonance, TEM—Transmission Electron Microscopy, ZP—Zeta Potential.
